# Temporo-parietal and fronto-parietal lobe contributions to theory of mind and executive control: an fMRI study of verbal jokes

**DOI:** 10.3389/fpsyg.2015.01285

**Published:** 2015-09-02

**Authors:** Yu-Chen Chan, Joseph P. Lavallee

**Affiliations:** ^1^Institute of Learning Sciences, National Tsing Hua UniversityHsinchu, Taiwan; ^2^International College, Ming Chuan UniversityTaipei, Taiwan

**Keywords:** humor, verbal jokes, logical mechanisms, theory of mind, executive function, fMRI, GTVH

## Abstract

‘Getting a joke’ always requires resolving an apparent incongruity, but the particular cognitive operations called upon vary depending on the nature of the joke itself. Previous research has identified the primary neural correlates of the cognitive and affective processes called upon to respond to humor generally, but little work has been done on the substrates underlying the distinct cognitive operations required to comprehend particular joke types. This study explored the neural correlates of the cognitive processes required to successfully comprehend three joke types: bridging-inference jokes (BJs), exaggeration jokes (EJs), and ambiguity jokes (AJs). For all joke types, the left dlPFC appeared to support common cognitive mechanisms, such as script-shifting, while the vACC was associated with affective appreciation. The temporo-parietal lobe (TPJ and MTG) was associated with BJs, suggesting involvement of these regions with ‘theory of mind’ processing. The fronto-parietal lobe (IPL and IFG) was associated with both EJs and AJs, suggesting that it supports executive control processes such as retrieval from episodic memory, self-awareness, and language-based decoding. The social-affective appreciation of verbal jokes was associated with activity in the orbitofrontal cortex, amygdala, and parahippocampal gyrus. These results allow a more precise account of the neural processes required to support the particular cognitive operations required for the understanding of different types of humor.

## Introduction

Successful humor achieves it effect by first generating a surprise through introducing an unexpected incongruity of some sort, and then amusement by providing the reader with the material needed to resolve the incongruity in a playful manner. A broad understanding of the cognitive operations required at the most general level, along with their neural correlates, has emerged in recent years. However, less work has been done in developing an account of the particular cognitive processes called upon by different types of humor, and even less has been done to identify the neural correlates associated with these cognitive processes. Attardo and Raskin’s General Theory of Verbal Humor (GTVH; Attardo and Raskin, 1991) provides perhaps the most comprehensive attempt to identify and categorize different types of verbal humor, along with the ‘logical mechanisms’ required for humor comprehension. Here, we adapt this theoretical framework to develop an account of the cognitive operations required for humor comprehension. Then, in the main part of this study, we attempt to identify the particular neural regions associated with these different cognitive operations.

The current study takes the ‘neural circuit model’ (NCM) of humor processing as its starting point (Chan et al., 2012, 2013). This model seeks to capture the neural correlates of the most general features of humor-related cognition, as delineated in Wyer and Collins’s (1992) comprehension-elaboration theory. The comprehension-elaboration theory divides humor processing into an initial ‘comprehension’ phase, during with the reader or listener first detects and then resolves an incongruity (Suls, 1972) and a subsequent ‘elaboration’ phase in which the implications of the humor are appreciated and the emotion of mirth or amusement results. In the NCM, the comprehension of verbal jokes starts with the identification of semantic incongruities through cognitive processes supported in the right middle temporal gyrus (MTG) and right medial frontal gyrus (MFG), followed by processes of semantic integration required to resolve the incongruity and re-establish coherence, supported by neural activity in the bilateral inferior frontal gyri (IFG), left superior frontal gyrus (SFG), and left inferior parietal lobule (IPL). Finally, the subsequent appreciation of, and affective response to the joke are associated with neural activity in the left ventromedial prefrontal cortex (vmPFC) and subcortical bilateral amygdalae and bilateral parahippocampal gyri (Chan et al., 2012, 2013; Chan, 2015).

These earlier studies did not take into account the different types of cognitive operations required to detect semantic incongruities and then to re-establish a coherent semantic understanding for different types of jokes. The present study thus seeks to extend this model by focusing on the neural regions associated with the different cognitive operations required to comprehend verbal jokes constructed using different *logical mechanisms*. The concept of logical mechanisms is drawn from GTVH (Attardo and Raskin, 1991). In the GTVH, verbal jokes are composed from six distinct knowledge resources (KRs). In common with many theories of humor processing, the GTVH first posits the presence of an incongruity, which in this theory is discussed in terms of a *script opposition. Scripts* refer to cognitive schemata or structures which contain and organize knowledge about the world (characteristics of people and situations, common routines, etc.) in long term memory. The text of any joke is at least partially compatible with two different scripts which are opposed in some manner. The joke’s punchline forces the reader to switch to the humorous script, often by making them realize that a different interpretation was possible from the very beginning. In the terms used earlier, detection of the incongruity marks awareness of a problem in interpreting the joke according to the initial, non-humorous script, and resolution of the incongruity occurs when the reader is able to call up the second, humorous script to re-establish a coherent understanding of the text. The GTVH further categorizes jokes using five additional KRs: the *logical mechanism* which logically connects the components of the joke in the humorous script, allowing the incongruity to be resolved; the situation within which the story takes place (including activities, objects, etc.); the *target* or ‘butt’ of the joke; the *narrative strategy* employed (dialog, riddle, story); and the particular *language* used in verbalizing the text. The present study focuses on the logical mechanisms. Logical mechanisms refer to the cognitive ‘rule’ or process (e.g., role exchange, analogy, juxtaposition) that must be implemented to resolve a joke’s incongruity so that comprehension of the joke can occur. Thus, focusing on these directs attention to the particular cognitive operations which must be performed in order to resolve the incongruity that is at the core of all jokes.

Attardo et al. (2002) attempted to provide a taxonomy of the different known types of logical mechanisms. The present study focuses on three of the logical mechanisms identified in that study: *inferring consequences*, *exaggeration*, and *juxtaposition*. In jokes constructed around the “inferring consequences” logical mechanism, a situation is presented in which either consequences are presented and the preceding events are to be inferred, or imminent consequences are left to be inferred from the details of the situation. In the second type, exaggeration, one or more qualities of an element within a script are exaggerated in some way and the resulting humor comes from the conceptual incongruity (Attardo et al., 2002; see also McGhee, 1979; Berger, 1993). Finally, where juxtaposition is used, two scripts are simply juxtaposed, often through simple linguistic ambiguity (as with puns; Attardo and Raskin, 1991).

This study makes use of three joke types, based on the above logical mechanisms. The first joke type used in the present study is ‘*bridging-inference.*’ Jokes were selected for this category if they were constructed using the ‘inferring consequences’ logical mechanism, with some action or personal characteristic implied but not made explicit in the text, and if the joke required the reader to make inferences to ‘bridge’ the ‘gap’ to construct the text coherently. Thus, in the golfer joke in **Table 1**, the setup leads the reader to access a script of a typical golfer from long term memory. In this script, the golfer is likely to eventually hit the ball, even if there are one or two misses. The punchline, however, leads the reader to generate a new script, based on the ant’s expectation that Jack will never hit the ball, and oppose it to the first one. In this new script, the humor lies in the opposition between a normal golfer and this disparaging portrait of Jack. Cognitively, inferencing is required to generate the new script, which is then opposed to the initial one.

**Table 1:** Sample verbal jokes and corresponding baseline stimuli.

Joke type (logical mechanism)	Joke		Non-joke (baseline)
		


	Setup	Punch line	Punch line
Bridging-inference (LM = Inferring Consequences)	Jack, a novice golfer, is out practicing. On his first shot, the ball lands near an anthill 10 yards away. He prepares for another shot, takes a swing and misses the ball entirely, killing a number of ants in the process. He tries again, with the same result. Not giving up, he prepares to take yet another swing.	An ant yells out: “Hurry, everyone! Climb onto the ball!”	An ant yells out: “Hurry, everyone! Get away from the hill!”
Exaggeration (LM = Exaggeration)	One day, Kevin went to a dentist. When he opened his mouth, the dentist said “Oh, your cavities are so deep. Oh, your cavities are so deep. Oh, your cavities are so deep.” Kevin wasn’t pleased, and said to the dentist “I know I have bad teeth, but you don’t need to say it three times!”	The dentist replied, “I didn’t. That was an echo.”	The dentist replied, “You must brush your teeth more frequently.”
Ambiguity (LM = Juxtaposition)	Boiled eggs were served for lunch at a kindergarten 1 day. Seeing an opportunity to teach her students about birth, the teacher picked up an egg, showed it to her students, and said: “Eggs were laid by hens. How did you come to be here?”	Children: “We came by the school bus.”	Children: “We came from our mom’s belly.”

*Exaggeration jokes* (EJs), constructed using the exaggeration logical mechanism, were defined as jokes in which some element of a situation was exaggerated in terms of degree or quantity to such an extreme as to violate common sense understanding. In the sample joke in **Table 1**, the punch line requires the reader to drastically revise the imagined size of Kevin’s cavities. The violation of expectations occurs because our initial script or schema contains normal size cavities incapable of generating echoes. We re-establish coherence by generating a script in which the patient has enormous cavities, and the opposition and the implications it makes apparent about the patient generate humor.

Finally, *ambiguity jokes* (AJs), constructed using the juxtaposition logical mechanism, were defined as jokes in which the humor is based on the juxtaposition of different possible interpretations which emerge as the joke is read or told. Linguistic jokes, relying on ambiguity and alternative interpretations (Attardo, 1994, 1997; Attardo et al., 1994; Oaks, 1994; Lew, 1997) and requiring disambiguation (Attardo, 2001; Goel and Dolan, 2001; Bekinschtein et al., 2011; Cheng et al., 2013; Chan, 2015), have been treated as a distinct type of joke in a number of studies (e.g., Schultz and Horibe, 1974). Thus, the sample joke in **Table 1** relies on two very different interpretations of “how did you come here” (interpretations which emerge more naturally in the original Chinese version). The boy’s response is incongruous with expectations generated by the first script, leading readers to return to the initial language used in the setup to generate a second script through another interpretation of the ambiguous language.

Based on the assertion of the GTVH model that different types of jokes are constructed around different logical mechanisms, we would expect that comprehending these different types of jokes requires different cognitive operations, supported by different neural regions. Bridging-inference jokes (BJs) rely on standpoint-shifting to make gap-filling inferences based on the perceived thoughts or intentions of characters in the joke. AJs and EJs both rely on linguistic and semantic operations. AJs require the construction of a different interpretation of the language in the setup of the joke. EJs, on the other hand, call for a semantic modification or distortion of the concepts called up by the language in the setup.

In this study, we hope to extend our earlier three-stage NCM of joke processing (Chan et al., 2012, 2013) to clarify the distinct cognitive and affective mechanisms underlying the processing of these different types of jokes (**Table 2**). In this study, then, event-related fMRI was used to measure neural activity while participants read BJs, EJs, and AJs, and corresponding non-joke baselines. We analyzed the main effects and interactions of stimulus type and funniness for each type. Additionally, conjunction analysis was performed to identify brain regions active across the contrasts.

**Table 2:** The three-stage neural circuit model of joke extended to distinguish between bridging-inference, exaggeration, and ambiguity jokes.

Three-stage	Common	Distinct		
		Bridging-inference jokes	Exaggeration jokes	Ambiguity jokes
Incongruity detection	All detect incongruities in jokes	(1) Semantic gap (MTG) (2) Belief-reasoning gap	(1) Semantic distortion (PFC) (2) Faulty reasoning (analogy)	(1) Semantic ambiguity (PFC) (2) Faulty reasoning
Incongruity resolution (logical mechanism)	All require semantic processing and schema shifting (dlPFC)	(1) Inferring consequences (TPJ) (2) Bridging-inferential mechanism (standpoint-shifting) (3) Bridging the gap and attributing intention others [theory of mind (ToM)]	(1) Exaggeration (IPL) (2) Sociolinguistic mechanism (linguistic-shifting) (3) From impossible to possible (retrieval of self-related episodic memory)	(1) Juxtaposition (IFG) (2) Disambiguation mechanism (linguistic-shifting) (3) Semantic conflict monitoring and selection
Humor elaboration	All elicit a feeling of amusement (vACC), but the intensity of such feeling varies	Affect regulation (OFC/vmPFC)	Linking reward to ironic exaggeration (amygdala)	Disambiguation under social bonding (parahippocampal gyrus)

The existing research allows us to put forward a limited number of predictions or hypotheses. In terms of BJs, we note that inferencing often, as in the sample joke in **Table 1**, requires attributing intentions to others, or theory of mind (ToM) ability. ToM has been shown to be associated with activity in the temporo-parietal junction (TPJ) by earlier research (Saxe and Kanwisher, 2003; Mason and Just, 2011), including earlier humor-related research (Samson et al., 2008, 2009; Vrticka et al., 2013 for review). Additionally, the MTG plays a special role in the identification of incongruities (Chan et al., 2013), and in the semantic processing required for joke comprehension generally (Goel and Dolan, 2001; Bartolo et al., 2006), but also for bridging inferences (Kim et al., 2012).

Previous humor-related studies have shown that the left IPL contributes to humor comprehension processing, including semantic integration and resolving incongruities (Chan et al., 2013; Shibata et al., 2014). The IPL [also known as ventral parietal cortex (VPC)] has been implicated in cognitive operations related to language, episodic memory encoding and retrieval, semantic association, and perceptual and motor reorienting (Cabeza et al., 2012 for review). It seems reasonable to expect that this region might be involved in comprehending exaggerated person or object features. Finally, earlier research has shown left IFG activation in response to semantically ambiguous humorous riddles (Bekinschtein et al., 2011). This region may be expected to play a role in the processing of AJs.

Reward activation association with jokes may contribute to reflecting the processing of incongruity between social interaction and expectations. Joke-induced reward processing has been shown to implicate cortical regions including the ventromedial prefrontal cortex (vmPFC) (e.g., Goel and Dolan, 2001; Chan et al., 2012) and subcortical regions including the amygdala (e.g., Mobbs et al., 2003; Watson et al., 2007; Bekinschtein et al., 2011; Chan et al., 2012), nucleus accumbens (e.g., Mobbs et al., 2003; Bekinschtein et al., 2011), and parahippocampal gyrus (PHG; e.g., Chan et al., 2012). The vmPFC/orbitofrontal cortex (OFC) may play a mediating role in coordinating the relationship between social affect and socially oriented intentions for BJs. Exaggeration, distortion, and ironic expression may enhance the perceived funniness of jokes, thus activating the amygdala for EJs. Finally, we also expect that the PHG would activate in recognition and reward-predicting for AJs.

## Materials and Methods

### Participants

Twenty-seven right-handed native Mandarin speakers (16 females) with no history of neurological or psychiatric problems participated in this study. Their ages ranged from 21 to 29 years (*M* = 24.11, *SD* = 2.17). The study was approved by the Research Ethics Committee of National Taiwan University and all participants gave their informed consent to participate before commencing the experiment.

### Stimuli

All jokes were written in Mandarin Chinese and designed to elicit humor-related cognitive and affective processes. The jokes used as stimuli were chosen by a group of six judges, all with experience in humor research. First, definitions were established for each joke type. To be classified as ‘ambiguous,’ jokes had to achieve their effect by being interpretable in more than one way. Inference jokes were defined as those in which some information required to comprehend and appreciate the humor was missing, requiring the reader/hearer to supply additional information through inference in order to understand the joke. Finally, exaggeration jokes were those that achieved their effect through an exaggerative distortion of features or aspects of the situation. Using these definitions, the group first performed a search for ambiguity, inferencing, and exaggeration from an existing joke corpus (Cheng et al., 2013; Chan, 2014) as well as from the internet, books and magazines. In this initial stage, 96 ambiguity jokes, 88 inferential jokes, and 63 exaggeration jokes were found.

In the second stage, each of these jokes were reviewed. Duplicates (jokes with significant overlap in content) were removed. Since gender differences have been found in the responses to jokes with sexual, political, ‘frightening’ or ‘disgusting’ content (Cheng et al., 2013), any jokes with such content were also removed. Jokes within each category were then examined. AJs were subdivided into jokes that were phonologically ambiguous, syntactically ambiguous, lexically ambiguous (semantically ambiguous at the level of word or phrase meaning), or semantically ambiguous (semantically ambiguous at the sentence level). For the present study, only semantically AJs were retained, and the phonologically, syntactically, and lexically AJs were removed. Of the remaining 66 AJs, the group chose the 60 felt to best represent the category.

The initial set of 63 EJs were next examined, with jokes including references to psychiatry (psychiatric hospitals, psychiatric patients, etc.) and nonsense jokes removed. (Psychiatry jokes were removed because, with this type of content, it is difficult to determine whether the humor derives from exaggeration or the ‘normal’ behavior of stereotypical psychiatric patients. Nonsense jokes were removed because, with such jokes, the humorous incongruity is typically not resolved or only partially resolved.) Of the 23 remaining EJs, the group selected the 20 that were felt to best represent the category.

The inferencing jokes were subdivided into jokes that required either backward or forward inferencing. Backward inferencing, or ‘bridging-inferential’ jokes were defined as those requiring the reader to make inferences about joke content in order to comprehend the joke, while forward inferencing jokes required the reader to make (typically disparaging) inferences about the characteristics of the target of the joke based on its content, in order to appreciate the humor. The backward inferencing or BJs were used in this study, and the forward inferencing jokes were removed. Of the 64 such jokes remaining in the corpus, the group selected the 60 felt to be most representative of the type for use as stimuli.

To ensure that the jokes were valid as stimuli, two behavioral pilot studies were conducted prior to the fMRI experiment. For the first pilot study, the 60 BJs and 60 AJs were used. Corresponding base-line trials were constructed by replacing the punch lines for all of these jokes with neutral (non-funny) stories of matching length and punctuation, resulting in 60 bridging-inference baseline stimuli (BS) and 60 ambiguous baseline stimuli (AS).

Sixty native Chinese-speaking participants (35 females, aged 22.17 ± 2.04 years, ranging from 19 to 29 years-old) were given the same standardized instructions and viewed each trial on a computer monitor. Participants rated each using E-Prime software on its degree of comprehensibility, funniness, and on whether it required inferencing in order to bridge a semantic gap. Ratings were made on a 9-point scale (1 = extremely incomprehensible/unfunny/no inferential bridging; 9 = fully comprehensible/funny/inferential bridging). In addition, participants also indicated whether they felt the jokes were ambiguous (1 = no, 2 = yes, 3 = not sure).

The mean and standard deviation for comprehensibility was 7.54 ± 1.13, indicating that all stimuli (joke and non-joke) were comprehensible to participants. The mean funniness rating for both joke types was 5.33 ± 1.39. A one-way repeated-measures ANOVA performed on participants’ funniness ratings was significant, *F*(3,177) = 29.13, *p* < 0.001, $\eta_{p}^{2}$ = 0.83, and Bonferroni *post hoc* tests revealed that the funny conditions were significantly funnier than the unfunny conditions. The mean rating for whether the jokes required inferencing to bridge a semantic gap was 4.70 ± 1.80. A one-way repeated-measures ANOVA was significant, *F*(3,177) = 17.15, *p* < 0.001, $\eta_{p}^{2}$ = 0.23, with BJs receiving significantly higher ratings than jokes in the other conditions. In term of ambiguity, the percentage of ‘no’ responses was 23.8%, ‘yes’ was 75.1%, and ‘not sure’ was 1.1% for the AJs. A Pearson’s Chi-square analysis revealed a significant relationship between stimuli type and ambiguous judgment, χ^2^(6) = 631.01, *p* < 0.001, indicating that there was a significant difference in the judged degree of ambiguity across the four conditions. The *post hoc* tests revealed that stimuli in the AJ condition (‘yes’ = 75.1%) were significantly more ambiguous than the AS (33.9%), BJs (35.1%), and BS (20.6%). Based on the results of this pilot study, we then selected the 30 most salient BJs and AJs.

To ensure that the EJs we chose would be funny, a second pilot study was conducted with a separate group of 48 native Chinese-speaking participants (30 females, 19–26 years-old, average age 21.75 ± 1.93 years) who viewed and rated the 30 BJs, 30 AJs, and 20 EJs and the corresponding baseline stimuli. Corresponding base-line stimuli were constructed by replacing the punch lines for all of these jokes with neutral (non-funny) stories of matching length and punctuation, resulting in 30 BS, 30 AS, and 20 ES (exaggeration baseline stimuli). Fewer EJs were used, as there were fewer suitable instances in our corpus of verbal jokes because, as noted above, the present study did not include psychiatry jokes and nonsense jokes.

Participants rated each trial on its degree of comprehensibility, funniness, need for backward-inferencing, exaggeration, and ambiguity on a 9-point scale (ranging from 1 = completely incomprehensible/unfunny/no inferencing required/not exaggerative/not ambiguous, to 9 = fully comprehensible/funny/requires inferencing/exaggerative/ambiguous; **Table 3**). A one-way repeated-measures ANOVA on participants’ bridging-inferential ratings was significant, *F*(5,235) = 38.61, *p* < 0.001, $\eta_{p}^{2}$ = 0.45, and Bonferroni *post hoc* tests revealed that the BJs were perceived as requiring significantly more backward-inferencing than the other five conditions. A one-way repeated-measures ANOVA on participants’ ambiguity ratings was significant, *F*(5,235) = 89.48, *p* < 0.001, $\eta_{p}^{2}$ = 0.65, and Bonferroni *post hoc* tests revealed that the AJs were significantly more ambiguous than those in the other five conditions. A one-way repeated-measures ANOVA on participants’ exaggerative ratings was significant, *F*(5,115) = 101.43, *p* < 0.001, $\eta_{p}^{2}$ = 0.82, and Bonferroni *post hoc* tests revealed that the EJs were significantly more exaggerative than those in the other five conditions.

**Table 3:** Mean and standard deviation for funniness, comprehensibility, bridging-inference, exaggeration, and ambiguity in six types of verbal stimuli.

Type	Funniness		Comprehensibility		Bridging-inference		Exaggeration		Ambiguity	
	*M*	*SD*	*M*	*SD*	*M*	*SD*	*M*	*SD*	*M*	*SD*
BJ	6.18	1.52	8.58	0.44	5.49	1.70	4.33	1.76	3.21	1.64
BS	2.55	1.27	7.95	1.01	3.50	1.51	3.38	1.34	2.40	1.18
EJ	5.69	1.77	8.48	0.54	4.75	1.67	7.95	0.79	2.96	1.50
ES	2.77	1.40	7.63	0.99	3.92	1.51	4.60	0.86	2.86	1.24
AJ	6.31	1.60	8.72	0.41	4.16	1.77	3.95	1.78	6.20	1.61
AS	2.72	1.31	8.14	0.80	3.37	1.47	2.78	1.20	3.48	1.44

*BJ, bridging-inference jokes; BS, bridging-inference baseline stimuli; EJ, exaggeration jokes; ES, exaggeration baseline stimuli; AJ, ambiguity jokes; AS, ambiguity baseline stimuli.*

A two-way repeated measures ANOVA with factors of 3 (type: bridging-inference, exaggeration versus ambiguity) × 2 (funniness: joke versus non-joke) was performed. There was a main effect of funniness, *F*(1,47) = 354.70, *p* < 0.001, indicating that jokes in the funny condition were significantly funnier than those in the unfunny conditions. There was a significant type and funniness interaction, *F*(2,94) = 14.18, *p* < 0.001. There was a simple main effect revealing that the EJs were significantly less funny than jokes of the other two types.

For the 80 jokes and 80 baseline stimuli, the setups were 75–95 characters in length (*M* = 83.88, *SD* = 6.65) and the punch lines were 15–20 characters in length (*M* = 17.84, *SD* = 1.92). Length and punctuation were matched across conditions for the setups and punch lines. Translated examples for all stimulus types are shown in **Table 1**.

### Experimental Paradigm

**FIGURE 1:**
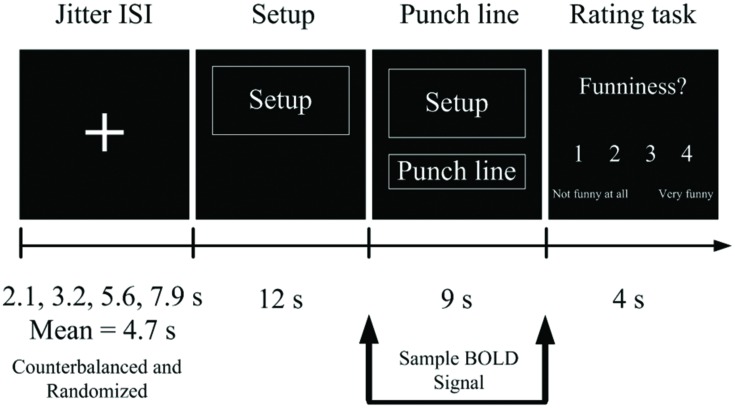
**An experimental trial timeline during the scanner.** Stimuli were presented in an event-related fMRI paradigm.

The experiment employed an event-related paradigm. The study investigated the distinct and shared neural correlates across three joke types (bridging-inference/exaggeration/ambiguity) and the funniness (joke/non-joke) contrast, resulting in six conditions. All stimuli were presented in black and white. While in the scanner, each participant was presented with 40 verbal jokes and 40 corresponding non-joke baseline stimuli. Within each trial, a jittered inter-stimulus interval (ISI) of 2.1, 3.2, 5.6, and 7.9 s was varied randomly and counterbalanced across events. The setup was shown once for 12 s, after which the punch line was delivered, lasting 9 s. Participants then provided a subjective funniness judgment by pressing one of four buttons on a keypad (1 = ‘not funny at all’ to 4 = ‘very funny’; **Figure 1**). The use of hand for the button-press responses was counterbalanced in the scanner. There were five functional runs in total. Trials in the six experimental conditions were pseudorandomized and counterbalanced across the five functional runs. A custom-built pseudorandom order list across conditions was generated using Matlab. Each functional run lasted 8 min and 4 s, with a 2-min break between runs. The total duration of the experiment was approximately 48 min and 6 s per participant.

### Image Acquisition

Functional images were acquired on a 3-tesla Megnetom Skyra Siemens MRI scanner using a 32-channel head coil to acquire a T2^∗^-weight gradient echo spiral pulse sequence sensitive to blood oxygenation level-dependent (BOLD) contrast. Visual stimuli were presented to the participants on a projector. Every volume contained thirty-two transversal slices of 4 mm thickness (no gap) in interleaved order that were oriented parallel to the anterior and posterior commissure (AC–PC) covering the whole brain with the following acquisition parameters: TE = 30 ms, TR = 2000 ms, flip angle = 90°, FOV = 240 mm × 240 mm, matrix size = 64 × 64, giving an in-plane spatial resolution of 3.75 mm × 3.75 mm × 4 mm. Each functional run acquiring 240 volumes took 8 m and 4 s. High-resolution T1-weighted structural images were also acquired using the 3D MPRAGE pulse sequence: TR = 1900 ms, TE = 3.30 ms, flip angle = 9°, 256 × 256 voxel matrix, FOV = 256 mm, 192 contiguous axial slices, thickness = 1.0 mm, and in-plane resolution: 1 mm × 1 mm × 1 mm.

### Image Analysis

Functional data were analyzed using SPM8 software (Statistical Parametric Mapping, Wellcome Department of Cognitive Neurology, London, UK). Following slice timing correction, and realignment, co-registered images were normalized to the standard Montreal Neurological Institute (MNI, McGill University, Montreal, QC, Canada) T1 template and the 3 mm × 3 mm × 3 mm voxel size of the written normalized images. The functional images were corrected for differences in slice-acquisition time to the middle volume and were realigned to the first volume in the scanning session using affine transformations. Statistical analyses were calculated on data that had been spatially smoothed using an 8-mm full-width-at-half-maximum (FWHM) Gaussian kernel with a high-pass filter (128-s cutoff period) to remove low frequency artifacts. The movement was no more than 3 mm in any plane.

For event-related analysis, the functions corresponding to the onset of different event types were constructed and convolved with a canonical hemodynamic response function (HRF) and its temporal derivative. In a first level analysis (single subject analyses), the different event types (BJ, BS, EJ, ES, AJ, and AS) were defined and parameter estimates for each regressor were calculated for each voxel. Stimuli were treated as individual events for analysis and modeled for the punch line using a canonical HRF. To increase the statistical sensitivity and to remove motion-related artifacts, we also included six motion parameters as regressors/nuisance covariates of no interest in the first level general linear model.

Individual contrast images were then entered into random-effects analysis (a second level) using the flexible factorial design. Therefore, analysis of the parametric modulation were analyzed using two-way analysis of variance (ANOVA), which allowed the study to parse the main effect of funniness (joke/non-joke) and the interaction between the two factors (type and funniness). Finally, conjunction analysis was performed to identify brain regions that were commonly active across the contrasts.

All reported areas of activation were considered significant at height (peak-level) threshold of *p* < 0.05 corrected for family-wise error rate (FWE) across the whole-brain for multiple comparisons at the voxel level with a cluster size greater than or equal to 20 voxels. To visualize the signal change for significant brain regions, time courses were extracted from the beta values of peak voxels of the regions. The SPM-MNI coordinates received from the statistical analysis were converted to Talairach coordinates with mni2tal conversion software (Talairach and Tournoux, 1988) and were obtained applying into labels of the corresponding brain regions (e.g., Brodmann areas).

## Results

### Behavioral Results

Participants were requested to rate their subjective funniness judgment on a 4-point scale (1 = not funny at all, 2 = not funny, 3 = funny, 4 = very funny) during the scanning procedure. In term of subjective ratings of joke and non-joke condition, the mean funniness ratings (Mean ± *SD*) for BJs, EJs, and AJs as well as corresponding baselines are shown in **Table 4**. A one-way repeated-measures ANOVA on participants’ funniness ratings was significant, *F*(5,130) = 162.10, *p* < 0.001, $\eta_{p}^{2}$ = 0.86, and Bonferroni *post hoc* tests revealed that the three joke conditions were significantly funnier than the three non-joke conditions.

**Table 4:** Mean, standard deviation, ratio, and overall score for funniness ratings during the scanning procedure.

Type	Funniness		Rating (ratio)					Overall	
	*M*	*SD*	1	2	3	4	Missing	Unfunny	Funny
BJ	3.07	0.42	4.4%	14.1%	51.1%	30.1%	0.2%	18.5%	81.2%
BS	1.86	0.36	35.3%	44.9%	16.3%	2.5%	1.0%	80.2%	18.8%
EJ	2.90	0.42	5.9%	20.7%	50.0%	23.0%	0.4%	26.6%	73.0%
ES	1.91	0.48	31.9%	48.5%	16.7%	3.0%	0.0%	80.4%	19.7%
AJ	3.12	0.42	1.7%	14.3%	53.8%	29.6%	0.5%	16.0%	83.4%
AS	1.88	0.39	35.1%	42.0%	19.8%	1.7%	1.5%	77.1%	21.5%

*(1) Stimulus types: BJ, bridging-inference jokes; BS, bridging-inference baseline stimuli; EJ, exaggeration jokes; ES, exaggeration baseline stimuli; AJ, ambiguity jokes; AS, ambiguity baseline stimuli. (2) Rating scales: 1 = not funny at all, 2 = not funny, 3 = funny, 4 = very funny. (3) Overall: Unfunny = 1 plus 2 ratings, Funny = 3 plus 4 ratings.*

A Pearson’s Chi-square analysis revealed a significant relationship between stimuli type and funniness judgment, χ^2^(20) = 855.50, *p* < 0.001, indicating that there is a difference in perceived funniness between the six conditions. The *post hoc* tests revealed that the jokes (BJs, EJs, and AJs) were significantly funnier than the non-joke baselines (BS, ES, and AS). There was no significant difference in the degree of funniness between the three joke types (BJs, EJs, and AJs), nor was there a significant difference between the ‘funny’ and ‘very funny’ ratings across the three joke types.

### fMRI Results for Distinct Neural Mechanisms

The present study performed different brain analyses of activations dissociated by different types of jokes. A two-way ANOVA revealed differences in brain activity for the main effects of funniness, and an interaction between type and funniness.

#### Main Effect of Funniness

The present study examined the main effect of funniness. The contrast of all jokes versus all baseline stimuli showed activation of a wide network of cortical and subcortical regions including the left ventral anterior cingulate cortex (vACC), bilateral TPJ, right amygdala, left middle occipital gyrus, and right frontoinsular (FI) region (**Table 5**).

**Table 5:** Brain regions for main effect of funniness (jokes versus non-jokes).

Region	BA	Side	MNI coordinates			*Z* score
			*x*	*y*	*z*	
Anterior cingulate cortex (ACC)	32	L	-3	41	4	7.56
Temporoparietal junction (TPJ)	39	L	-54	-69	25	6.85
Temporoparietal junction	39	R	57	-61	25	5.75
Amygdala	–	R	24	-4	-17	5.28
Middle occipital gyrus	19	L	-36	-85	1	5.21
Frontoinsular (FI)	13	R	30	26	1	4.62

*MNI, Montreal Neurological Institute; L, left; R, right; BA, Brodmann’s area. Activation threshold was set at p < 0.05 FWE (family-wise error rate) corrected for multiple comparisons at the peak level for the whole brain.*

#### Interaction between Type and Funniness

The interaction between type and funniness revealed activation in the right middle frontal gyrus (MNI coordinates: 33, 26, 46; MFG, BA 8; *Z* = 4.99), and left posterior cingulate cortex (MNI coordinates: 0, -55, 19; PCC, BA 23; *Z* = 4.82).

#### Simple Main Effect for Each Type

A *post hoc* test showed significant simple main effects for each of the different types.

##### Bridging-Inference Jokes (BJs) versus Non-Joke Baseline (BS)

**FIGURE 2:**
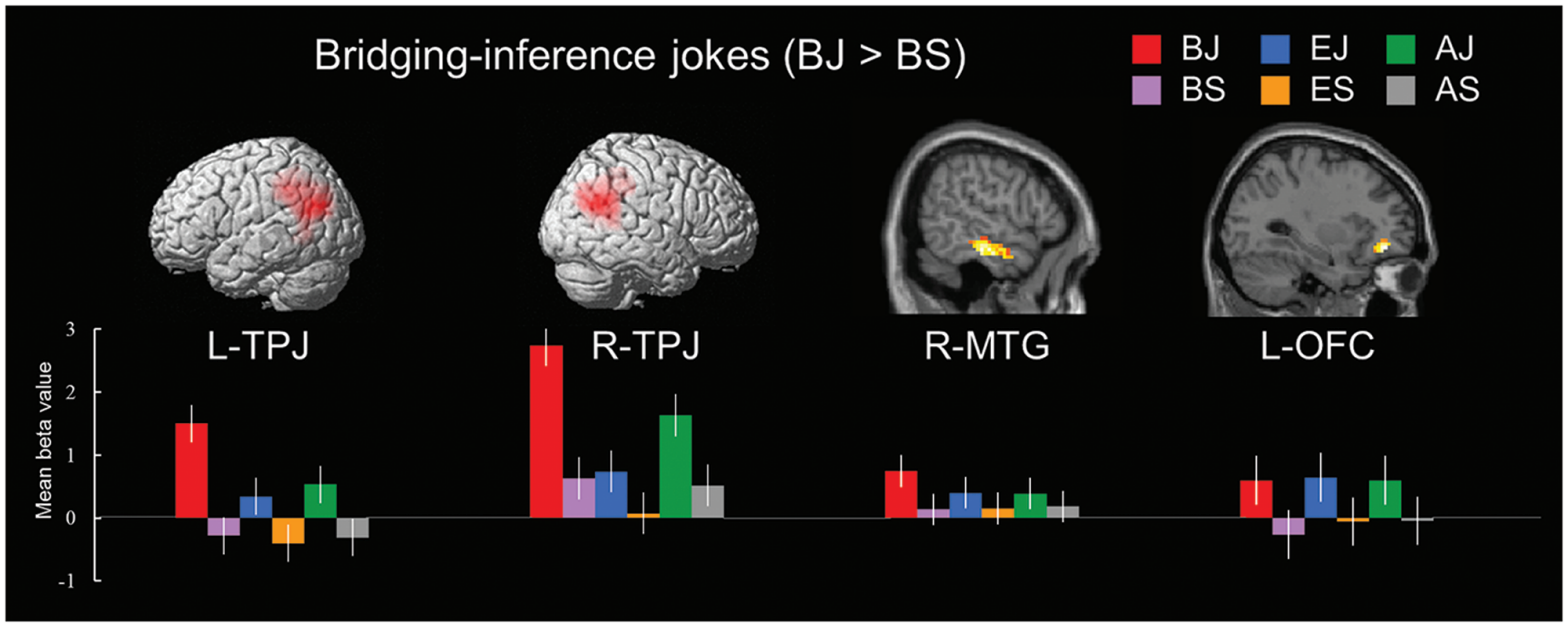
**Distinct neural mechanisms for bridging-inference jokes in temporo-parietal lobe (TPJ and MTG). (Top)** Brain images of greater activations were found for simple main contrast of bridging-inference jokes with corresponding non-joke baseline (BJ-BS) in TPJ and MTG during cognitive processing and in OFC during affective processing. MNI coordinates for distinct regions can be found in **Table 6**. **(Bottom)** Bars show mean beta values of peak voxels for each of the three types. Error bars represent standard error of the mean (SEM). L, left; R, right; TPJ, temporoparietal junction; MTG, middle temporal gyrus; OFC, orbitofrontal cortex; BJ, bridging-inference jokes; BS, bridging-inference baseline stimuli; EJ, exaggeration jokes; ES, exaggeration baseline stimuli; AJ, ambiguity jokes; AS, ambiguity baseline stimuli.

In the bridging-inference type condition, the BJs versus BS contrast revealed a network of cortical regions involved in the process of inferring consequences. Significant activations were found in the bilateral TPJ (BA 39), left vACC (BA 32), right MTG (BA 21), and left OFC (BA 11; **Table 6** and **Figure 2**).

**Table 6:** Brain regions differentially activated for the simple main effects of joke type.

Region	BA	Side	MNI coordinates			*Z* score
				
			*x*	*y*	*z*	
**Bridging-inference jokes versus baselines**				
Temporoparietal junction	39	L	-54	-69	25	7.80
Ventral anterior cingulate cortex	32	L	-3	41	4	6.75
Temporoparietal junction	39	R	57	-61	25	6.66
Middle temporal gyrus	21	R	57	-22	-20	5.08
Orbitofrontal cortex (OFC)	11	L	-30	41	-11	4.80
**Exaggeration jokes versus baselines**				
Amygdala	–	R	27	-10	-14	6.03
Claustrum	–	L	-30	5	10	5.90
Inferior parietal lobule	40	L	-60	-40	40	5.65
Inferior parietal lobule	40	R	66	-37	34	4.87
Inferior frontal gyrus	46	R	42	41	7	4.74
**Ambiguity jokes versus baselines**				
Ventral anterior cingulate cortex	24	L	-3	41	-8	6.49
Parahippocampal gyrus	35	R	24	-22	-17	4.66

*MNI, Montreal Neurological Institute; L, left; R, right; BA, Brodmann’s area. Activation threshold was set at *p* < 0.05 FWE (family-wise error rate) corrected for multiple comparisons at the peak level for the whole brain.*

##### Exaggeration Jokes (EJs) versus Non-Joke Baseline (ES)

**FIGURE 3:**
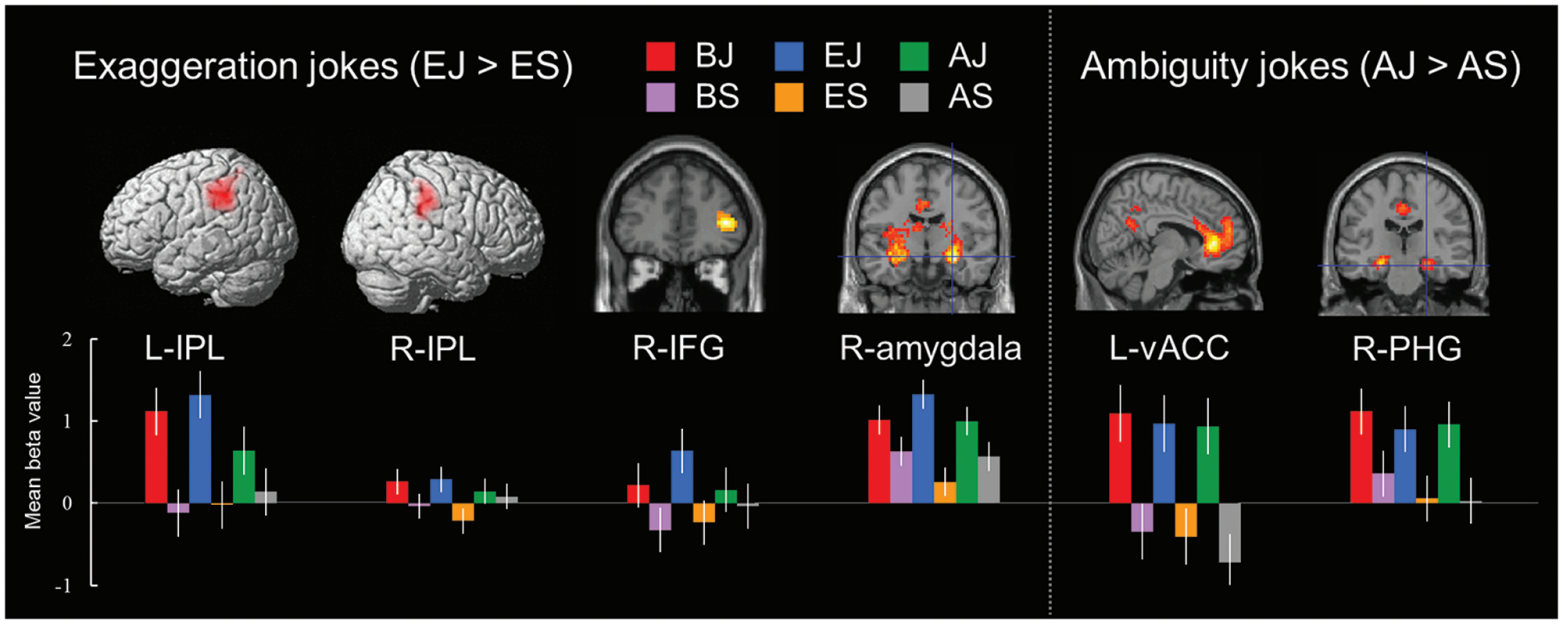
**Distinct neural mechanisms for exaggeration jokes and ambiguity jokes in fronto-parietal lobe (IPL and IFG). (Left top)** Brain images of greater activation were found for simple main contrast of exaggeration jokes with corresponding non-joke baseline (EJ > ES) in bilateral IPL and right IFG during cognitive processing and in right amygdala during affective processing. **(Right top)** Brain images of greater activation were found for simple main contrast of ambiguity jokes with corresponding non-joke baseline (AJ > AS) in left vACC and right PHG during affective processing and extending to ventrolateral prefrontal cortex (IFG) and frontopolar cortex (MFG) during cognitive processing. MNI coordinates for distinct regions can be found in **Table 6**. **(Bottom)** Bars show mean beta values of peak voxels for each of the three types. Error bars represent SEM. L, left; R, right; IPL, inferior parietal lobe; IFG, inferior frontal gyrus; vACC, ventral anterior cingulate cortex; PHG, parahippocampal gyrus.

In the exaggeration type condition, the EJs versus ES contrast also revealed a network of cortical and subcortical regions presumably underlying comprehension of exaggerated elements. Significant activations were found in the right amygdala, left claustrum, bilateral IPL, and right IFG (**Table 6** and **Figure 3**). Activation in the claustrum extended to the left vACC (BA 24, Z = 5.78) and left frontopolar cortex (MFG, BA 10, Z = 4.91).

##### Ambiguity Jokes (AJs) versus Non-Joke Baseline (AS)

In the ambiguity type condition, the AJs versus AS contrast revealed a network of cortical and subcortical regions involved in disambiguation. Significant activation was found in the left ACC, centering in the vACC. Significant activation was also found in the right PHG (**Table 6** and **Figure 3**). Activation in both the left vACC and right PHG extended to the frontopolar cortex (FPC, BA 10) and ventrolateral prefrontal cortex (vlPFC, BA 47/46) including bilateral anterior regions of the rostral medial frontal cortex (arMFC, Z = 5.66/4.80) and the left IFG (Z = 4.18).

#### Regions Differentially Engaged by Bridging-Inference Jokes during Incongruity-Resolution Processing

**FIGURE 4:**
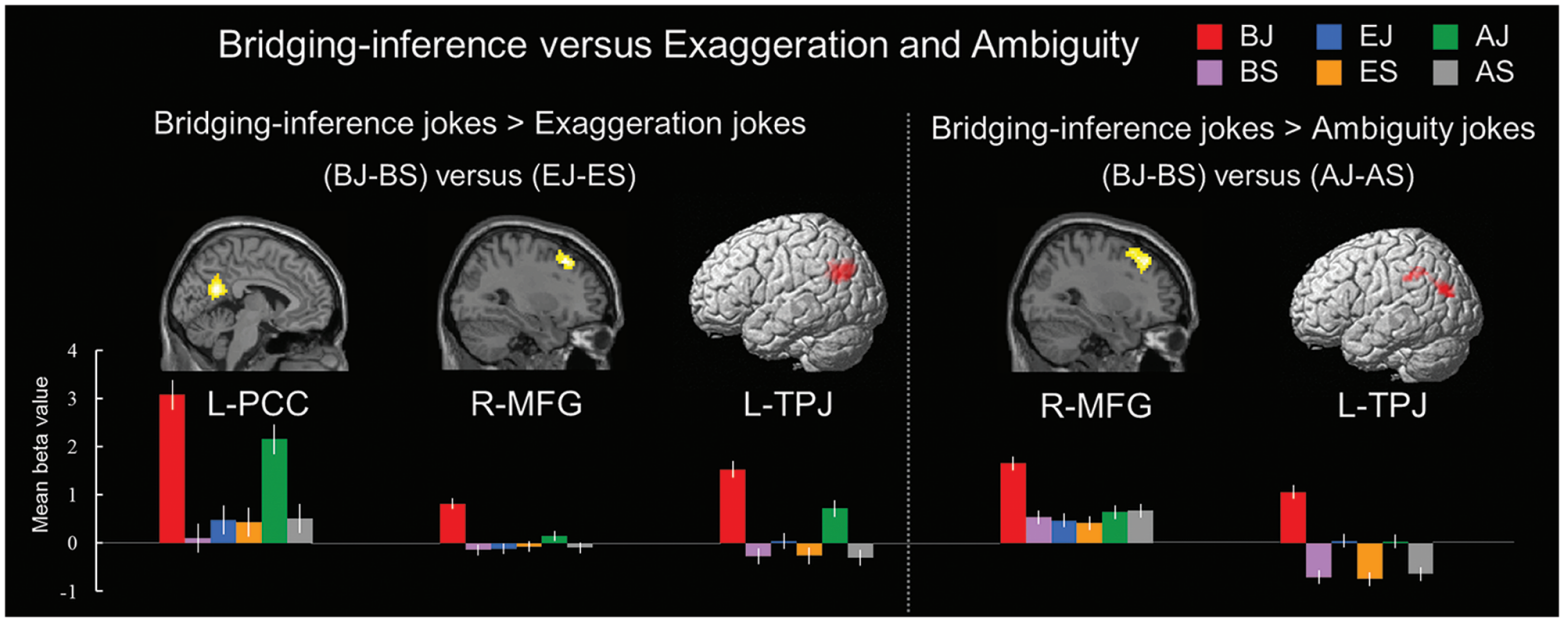
**Distinct neural mechanisms for bridging-inference jokes versus exaggeration jokes and bridging-inference jokes versus ambiguity jokes. (Left top)** Brain images of greater activations were found for contrast of bridging-inference jokes with exaggeration jokes [(BJ-BS) > (EJ-ES)] in PCC, MFG, and TPJ. **(Right top)** Brain images of greater activations were found for contrast of bridging-inference jokes with ambiguity jokes [(BJ-BS) > (AJ-AS)] in MFG and TPJ. **(Bottom)** Bars show mean beta values of peak voxels. Error bars represent SEM. L, left; R, right; PCC, posterior cingulate cortex; MFG, middle frontal gyrus; TPJ, temporoparietal junction.

A comparison of neural activation associated with viewing BJ-BS versus EJ-ES revealed significant activation in the left PCC, right MFG, and left TPJ. In addition, the BJ-BS versus AJ-AS contrast revealed activation in the right MFG and left TPJ (**Table 7** and **Figure 4**). The reverse contrast between exaggeration and bridging-inferencing revealed no significant areas of activity. The reverse contrast between ambiguity and bridging-inferencing also revealed no significant areas of activity.

**Table 7:** Regions differentially engaged and commonly recruited.

Region	BA	Side	MNI coordinates			*Z* score
				
			*x*	*y*	*z*	
**(A) Regions differentially engaged**				
*Bridging-inference jokes > exaggeration jokes*						
Posterior cingulate cortex	31	L	0	-58	22	5.19
Middle frontal gyrus	8	R	30	23	46	4.88
Temporoparietal junction	39	L	-54	-67	28	4.76
*Bridging-inference jokes > ambiguity jokes*						
Middle frontal gyrus	8	R	30	32	46	5.05
Temporoparietal junction	39	L	-51	-69	25	4.65
**(B) Regions commonly recruited**				
Dorsolateral prefrontal cortex (dlPFC)	9	L	-9	44	25	5.69
Ventral anterior cingulate cortex	24	L	-3	29	7	5.04

*Activation threshold was set at p < 0.05 FWE (family-wise error rate) corrected for multiple comparisons at the peak level for the whole brain. (A) Regions activated for bridging-inference jokes but not exaggeration jokes and ambiguity jokes discovery was revealed by subtraction. (B) Brain regions commonly activated for the three types of jokes, as revealed by a conjunction analysis.*

### fMRI Results for Common Neural Mechanisms

**FIGURE 5:**
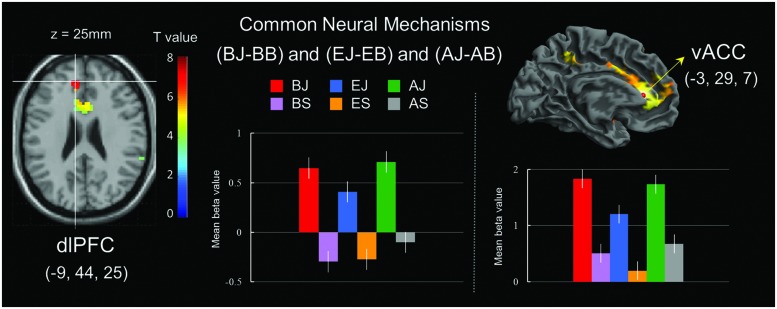
**Common neural mechanisms of general joke processing. (Left)** Commonly activated region of the left dorsolateral prefrontal cortex (dlPFC) in joke comprehension stage. **(Right)** Commonly activated region of the ventral anterior cingulate cortex (vACC) in joke appreciation stage. Bars show mean beta values of peak voxels for the left dlPFC (BA 9) and vACC (BA 32). Error bars represent SEM.

A conjunction analysis of processing for bridging-inference (BJ-BS), exaggeration (EJ-ES), and ambiguity (AJ-AS) jokes revealed common regions in the left dorsolateral prefrontal cortex (dlPFC; presumably related to cognitive processing) and vACC (presumably associated with affective processing; **Table 7** and **Figure 5**). Activation for the vACC spread to the midbrain (74 voxels), lentiform nucleus (486 voxels), putamen (376 voxels), and thalamus (273 voxels).

## Discussion

Incongruity is necessary for humor, and these incongruities are resolved based on logical mechanisms which recur for particular types of jokes. The present study showed an interaction between stimulus type and funniness, with evidence for distinct neural substrates underlying processing for different logical mechanisms associated with three types of verbal jokes.

In BJs, the temporo-parietal lobe (TPJ and MTG) were found to be active. Bilateral TPJ regions were more active for BJs, indicating that the TPJ is involved in resolving incongruities in cases where making inferences about the thoughts of others (ToM) is required. Other active regions of the MTG likely associated with the incongruity detection and semantic processing required to ‘get’ the joke. ToM is critical in attributing intentions to one’s own and to others’ actions. Although previous neuroimaging studies have indicated that the frontal lobes support the ability to process others’ mental states (Cabeza et al., 2012 for a review; Fletcher et al., 1995; Gallagher et al., 2000), Samson et al. (2004) have also found that the TPJ is necessary for reasoning about the beliefs of others, suggesting that the TPJ sustains not only low-level social perception, but also higher-level social reasoning. Patients with damage to the TPJ were also found to have difficulty in attributing intentions in a belief-reasoning task (Apperly et al., 2004). Earlier humor-related research also showed that the TPJ plays a special role in social situations involving inferring the intention underlying the behavior of others (Samson et al., 2008, 2009; Vrticka et al., 2013). Our results thus appear consistent with these earlier results on temporal-parietal lobe contributions to ToM. Social affective functions have also been associated with activity in the OFC (Goel and Dolan, 2007) and our results are consistent with the existence of a ‘mirror circuit’ in which the TPJ and OFC contribute to the attribution of mental states to others in TPJ and to the felt experience of socially related amusement in the OFC.

Exaggeration jokes showed particular activation in the parietal lobe (bilateral IPL) and the frontal lobe (right IFG, BA 46). The fronto-parietal network, which consists of the vlPFC in IFG (BA 46/47) and regions in the IPL, has been supposed to be engaged in attentional networks, especially in detection and resolution of rare, salient, and unexpected events (see Corbetta and Shulman, 2002, for a review). These ventral fronto-parietal areas have been associated with a number of attention-related processes related to sustaining attention or vigilance, suggesting that the IPL plays a crucial role in salience detection and phasic alerting (Singh-Curry and Husain, 2009), in detecting feature-driven or bottom up attentional processes, especially in evaluating stimuli as novel or highly significant (Bledowski et al., 2004). The rostral prefrontal cortex (rostral PFC) or frontopolar cortex (FPC) in the MFG (BA 10) has been associated with mentalizing activity related to social functions (Gilbert et al., 2007) and detecting the degree of novelty in both environmental stimuli and self-generated representations (Burgess et al., 2007). In terms of joke processing, it seems likely that the IPL is involved in detecting and resolving events which are particularly rare, novel or salient, while the frontopolar cortex (FPC, BA 10) might be more involved in detecting and evaluating unexpectedly exaggerated features. These ventral fronto-parietal areas also have been associated with a number of executive control processes related to working memory (Naghavi and Nyberg, 2005), decision making (Andersen and Cui, 2009), resisting interference to one’s own perspective (Ruby and Decety, 2003), and shifting perspective or maintaining a separation between alternative perspectives (Gallagher and Frith, 2003).

These regions have also been implicated as part of the ‘mirror circuit’ used to distinguish representations of self from representations of others (Decety and Sommerville, 2003). The right frontal lobe may be unique in integrating cognitive and affective information (Shammi and Stuss, 1999). The right ventral frontal cortex (VFC) has been implicated in a role of episodic memory retrieval (Henson et al., 1999), and generation and maintenance in set-shift transformations (Goel and Vartanian, 2005). The activation of these regions may be associated with the participants focusing on making alterations to their internal representations (scripts), to reflect the situation revealed in the punch line from their own perspective.

Previous investigations have suggested that the amygdala contributes to the experience of positive reward and is a key component of the dopaminergic reward system in humor appreciation (e.g., Mobbs et al., 2003; Bartolo et al., 2006; Watson et al., 2007; Bekinschtein et al., 2011; Chan et al., 2012; Chan, 2015). The dlPFC modulates the amygdala, which suggests that affective reappraisal involves a pathway linking cortical and subcortical regions in emotion regulation (Ochsner et al., 2012). The present study may thus be consistent with the idea that surprise, distortion, and ironic expression enhance the perceived funniness of EJs via the amygdala.

Finally, the processing of AJs requiring semantic disambiguation resulted in activation in the left vACC and the right PHG, extending to the frontopolar cortex (MFG, BA 10) and vlPFC (IFG, BA 47/46). The ACC in the human brain is known to contain von Economo neurons (Watson et al., 2007; Weems, 2014). The vACC plays a role in emotion appraisal and regulation (Phillips et al., 2003). Previous studies have found that the vACC (Kohn et al., 2011), PHG (Amir et al., 2015), and vlPFC (Hoffman et al., 2010) contribute first to executive control processes related to semantic processing and then to the felt experience of amusement. The findings of the present study related to the processing of AJs thus appears to be consistent with this earlier research.

The present study found a type-funniness interaction in the right MFG (BA 8) and left PCC (BA 31), indicating that type (bridging-inferencing, exaggeration, and ambiguity) and funniness (joke/non-joke) evoked different responses. The joke endings were unexpected and required script-shifting processes that were not necessary in processing the non-joke endings. The MFG (i.e., dlPFC) may play a key role in this process of script shifting. The PCC has been found to play a role in emotional processing (Maddock et al., 2003), empathy (Völlm et al., 2006), and ToM (Fletcher et al., 1995), perhaps specifically associated with the social cognitive processing common in jokes. Thus, the results for the interaction seem to reflect the particular cognitive and affective processes associated with humor comprehension and appreciation.

Furthermore, the present study also found that the dlPFC and vACC were active for all three joke types. The dlPFC region likely supports the cognitive operations required across joke types, such as script-shifting (Azim et al., 2005; Chan, 2015 for review; Coulson, 2001; Uekermann et al., 2007). In addition, vACC activation presumably reflects a common affective mechanism for joke appreciation and the feeling of amusement for all three joke types (Chan et al., 2012; Chan, 2015).

Our earlier research, developing a three-stage NCM of humor processing, found that the MTG and MFG were activated during incongruity detection and that the IPL, IFG, and SFG were activated during incongruity resolution (Chan et al., 2012, 2013). The present study found that during incongruity detection, MTG activation was particularly associated with BJs and that frontopolar cortex (MFG, BA 10) activation was associated with both EJs and AJs. Incongruity resolution was associated with TPJ activation for BJs and IPL and IFG activation for EJs and AJs.

The three-stage NCM additionally asserted that the vmPFC, amygdala, and PHG were likely responsible for the affective response to humor during the elaboration stage. The present study further distinguishes the neural mechanisms associated with different types of jokes. During the elaboration stage, when humor appreciation occurs with its social and affective components, activation was found to occur in the OFC/vmPFC (BA 11) for BJs, the amygdala for EJs, and in the PHG for AJs.

To summarize, the temporo-parietal lobe (TPJ and MTG) was specifically involved in BJs, probably reflecting the importance of ToM processing for this type of joke. The fronto-parietal lobe (IPL and IFG) regions were particularly active for EJs and AJs. In addition, the left dlPFC appears to subserve a common cognitive mechanism required for understanding all types of jokes, whereas the vACC appears to play a common role in affective appreciation. Future studies might further examine the functional connectivity between these regions, and extend this approach to other logical mechanisms associated with particular joke types.

## Conflict of Interest Statement

The authors declare that the research was conducted in the absence of any commercial or financial relationships that could be construed as a potential conflict of interest.
